# Identification of LINC00173 in Myasthenia Gravis by Integration Analysis of Aberrantly Methylated- Differentially Expressed Genes and ceRNA Networks

**DOI:** 10.3389/fgene.2021.726751

**Published:** 2021-09-16

**Authors:** Si Xu, Tianfeng Wang, Xiaoyu Lu, Huixue Zhang, Li Liu, Xiaotong Kong, Shuang Li, Xu Wang, Hongyu Gao, Jianjian Wang, Lihua Wang

**Affiliations:** Department of Neurology, The Second Affiliated Hospital of Harbin Medical University, Harbin, China

**Keywords:** lncRNA, ceRNA, myasthenia gravis, biomarker, network

## Abstract

Myasthenia gravis (MG) is an autoimmune disease associated with autoantibody production that leads to skeletal muscle weakness. The molecular mechanisms underlying MG are not fully understood. We analyzed the gene expression profile (GSE85452) and methylation profile (GSE85647) of MG samples from the GEO database to identify aberrantly methylated-differentially expressed genes. By integrating the datasets, we identified 143 hypermethylation-low expression genes and 91 hypomethylation-high expression genes. Then we constructed PPI network and ceRNA networks by these genes. Phosphatase and tensin homolog (PTEN) and Abelson tyrosine-protein kinase (ABL)1 were critical genes in both PPI networks and ceRNA networks. And potential MG associated lncRNAs were selected by comprehensive analysis of the critical genes and ceRNA networks. In the hypermethylation-low expression genes associated ceRNA network, sirtuin (SIRT)1 was the most important gene and the lncRNA HLA complex (HC) P5 had the highest connection degree. Meanwhile, PTEN was the most important gene and the lncRNA LINC00173 had the highest connection degree in the hypomethylation-high expression genes associated ceRNA network. LINC00173 was validated to be upregulated in MG patients by qRT-PCR (*P* = 0.005), which indicated LINC00173 might be a potential biomarker for MG. These results provide a basis for future studies on the molecular pathogenesis of MG.

## Introduction

Myasthenia gravis (MG) is an autoimmune disease that involves cellular immunity and complement activation and is characterized by impaired transmission at the neuromuscular junction, leading to skeletal muscle weakness and autoantibody production. The disease is mainly caused by the loss of immune tolerance, which induces the production of antibodies mainly against acetylcholine receptor (AChR), triggering an abnormal immune response (Gilhus and Verschuuren, 2015). The underlying cause of MG is unclear, but genetic factors may determine an individual’s disease risk. For example, the association between human leukocyte antigen (HLA) class I and II genes and MG is well established (Vandiedonck et al., 2005). MG-associated susceptibility genes such as those encoding cytotoxic T lymphocyte-associated protein (CTLA)-4, tumor necrosis factor (TNF)-α, protein tyrosine phosphatase non-receptor type (PTPN)22, and interleukin (IL)-10 have been implicated in other autoimmune diseases (Avidan et al., 2014).

In addition to the abovementioned genes, environmental factors such as pollutants, drugs, and pathogens are thought to increase the risk of MG (Cavalcante et al., 2013). Epigenetic modifications play an important role in gene regulation; they include DNA methylation, chromatin remodeling, non-coding RNA, and histone modifications. DNA methylation involves the addition of a methyl group to the 5th carbon position of cytosine at CpG dinucleotides, which are typically found in gene promoters; this prevents transcription factors from binding, thereby inhibiting gene expression (Schultz et al., 2015). CTLA4 methylation may promote the development of MG by increasing cytokine expression through upregulation of autoantibodies against AChR and erythrocyte acetylcholinesterase (Fang et al., 2018). Growth hormone secretagogue receptor (GHSR) hypermethylation was observed in thymoma samples of MG patients, especially in late stages of disease (Coppede et al., 2020). It was also reported that G0/G1 switch (G0S)2 was upregulated in B cells and CD8^+^ T cells of AChR-positive MG patients, but this was significantly attenuated by G0S2 methylation (Xu et al., 2020). These findings suggest that gene methylation contributes to the pathogenesis of MG.

Non-coding (nc) RNAs such as micro (mi) RNAs and long (l) ncRNAs modulate the expression of protein-coding genes in a variety of biological processes and play a key role in the occurrence of MG (Jonas and Izaurralde, 2015; Chen et al., 2017). For example, members of the lethal (let)-7 lncRNA family were downregulated in peripheral blood mononuclear cells (PBMCs) of MG patients, and the level of let-7c was negatively correlated with that of IL-10 (Jiang et al., 2012). Moreover, the lncRNA interferon gamma (IFN-γ) antisense RNA (IFNG-AS)1 was found to be differentially expressed in MG patients and negatively regulated the expression of HLA-DRB and HLA-DOB (Luo et al., 2017). The competing endogenous (ce) RNA theory states that transcripts sharing common miRNA binding sites compete to bind identical miRNAs for mutual regulation of expression levels (Salmena et al., 2011). Moreover, mRNAs, lncRNAs, and other types of RNA can adsorb miRNAs through miRNA binding elements and thereby affect their function. LncRNAs are endogenous sponges that regulate miRNA activity (Wang et al., 2015). The lncRNA small nucleolar RNA host gene (SNHG)16 was shown to regulate the expression of IL-10 by sponging let-7c-5p as a ceRNA in MG (Wang J. et al., 2020); and metastasis-associated lung adenocarcinoma transcript (MALAT)1, acting as a ceRNA for the miRNA miR-338-3p, directly induced male-specific lethal complex subunit (MSL)2 expression in MG (Kong et al., 2019). However, it remains unclear whether aberrantly methylated genes are related to ceRNAs in MG.

To answer this question, in this study, we analyzed the gene expression and methylation profiles of MG samples from the Gene Expression Omnibus (GEO) database. Hypermethylated genes with low expression (hypermethylation-low expression genes) and hypomethylated genes with high expression (hypomethylation-high expression genes) were identified by integrating differentially expressed genes (DEGs) and differentially methylated genes (DMGs). Functional enrichment analysis and protein–protein interactions (PPI) were investigated by bioinformatic methods. Based on the results, we constructed an aberrantly methylated-differentially expressed genes associated ceRNA network (AMCEN) for MG and selected potential biomarkers by comprehensive analysis of critical genes and ceRNA networks. This study may provide molecular-level insight into the pathogenesis of MG.

## Materials and Methods

### Data Sources

Gene expression (GSE85452) and gene methylation (GSE85647) datasets for MG were obtained from the GEO.^1^ GSE85452 (platform: GPL10558 HumanHT-12 v4.0 Expression BeadChip; Illumina, San Diego, CA, United States) was based on monocytes from 10 MG patients and 9 controls; and GSE85647 (platform: GPL13534 HumanMethylation450 BeadChip; Illumina) was based on monocytes from 9 MG patients and 7 controls. In order to avoid the influence of genomic similarity of monozygotic twin in the datasets on the experimental results, if monozygotic twins in the datasets were not all MG patients, they were excluded from the analysis. We manually searched PubMed^2^ for MG risk miRNAs in articles published before December 1st, 2020 using the keywords “microRNA” or “miRNA” or “miR” and “MG,” with the species limited to “Homo sapiens.” We then manually selected miRNAs that met the following criteria: (i) differentially expressed with statistical significance between MG patients and healthy controls; and (ii) detected by reliable experimental methods (microarray, real-time PCR, etc.). Experimentally verified mRNA–miRNA interactions were downloaded from miRTarBase (release 7.0) (Chou et al., 2018); the data were supported by luciferase reporter assay or western blotting. miRNA–lncRNA interactions were obtained from starBase v3.0 (Li et al., 2014), DIANA-LncBase v2.0 (Paraskevopoulou et al., 2013), and LncACTdb v2.0 (Wang et al., 2019) which contain interactions that have been high-throughput experimentally validated (e.g., HITS-CLIP, PAR-CLIP, iCLIP, CHIP, and CLASH).

### Data Processing

We used GEO2R online software^3^ to analyze expression data for MG patients and healthy controls. For the GES85452 and GSE85647 datasets, *P* < 0.05 and | t| > 2 was used as the cutoff criteria to identify DEGs and DMGs. Hypermethylation-low expression genes were obtained by overlapping hypermethylated and downregulated genes, and hypomethylation-high expression genes were obtained by overlapping hypomethylated and upregulated genes. DMG–miRNA interactions were determined by matching hypermethylation-low expression genes or hypomethylation-high expression genes with mRNA–miRNA interaction data from miRTarBase (release 7.0). We then identified mRNA–miRNA–lncRNA ceRNA triples by overlapping miRNAs from DMG–miRNA and miRNA–lncRNA interaction datasets. To increase the reliability of the data, the manually selected MG-associated miRNAs were also matched with the mRNA–miRNA–lncRNA ceRNA triples to obtain MG-associated aberrantly methylated-differentially expressed involved ceRNA interactions.

### Functional Enrichment Analysis

We performed functional enrichment analysis of hypermethylation-low expression and hypomethylation-high expression genes with the Database for Annotation, Visualization and Integrated Discovery (DAVID) platform^4^ (Dennis et al., 2003). Gene Ontology (GO) analysis included biological process, cellular component and molecular function terms (Gene Ontology, 2006); and we also conducted a Kyoto Encyclopedia of Genes and Genomes (KEGG) pathway enrichment analysis (Kanehisa and Goto, 2000). *P* < 0.05 was set as the cutoff for statistical significance.

### Analysis of GSEA Enrichment

We next performed GSEA analysis to avoid missing the genes which actually play an important part during the process of identifying differential expressed genes among the data. Gene set permutations were performed 1,000 times for each analysis. The normalized enrichment score (NES) > 1 and nominal *P*-value < 0.05 were regarded as significant.

### PPI Network Construction and Module Analysis

The construction of PPI networks is important for elucidating the molecular mechanisms underlying cellular activities. We used the Search Tool for the Retrieval of Interacting Genes (STRING)^5^ database and Cytoscape v3.8.1^6^ to construct PPI networks of hypermethylation-low expression and hypomethylation-high expression genes, respectively. An interaction score of 0.4 was taken as the cutoff. The Molecular Complex Detection (MCODE) plugin of Cytoscape software was used to define modules in the PPI network with degree cutoff = 2, node score cutoff = 0.2, k-core = 2, and maximum depth = 100. Hub genes were selected with cytoHubba based on a connection degree > 10.

### Cumulative Hypergeometric Test

Aberrantly methylated mRNA–lncRNA ceRNA interaction pairs sharing the same miRNA were identified by cumulative hypergeometric testing (Zhang G. et al., 2018). The *P*-value for each interaction pair was computed using the following formula:


P=1-∑k=0x(mk)⁢(N-mn-k)(Nn)

where N is the total number of miRNAs from DMG–miRNA interactions; n and m are the number of miRNAs associated with 1 mRNA and 1 lncRNA, respectively, and x is the number of miRNAs shared by the mRNA and lncRNA. Interactions of aberrantly methylated mRNA–lncRNA ceRNA pairs with a *P*-value < 0.05 were considered significant.

### LncRNA–mRNA Co-expression Analysis

We performed a co-expression correlation analysis of lncRNA–mRNA interaction pairs by calculating the Pearson correlation coefficient (PCC) of lncRNA and mRNA expression data downloaded from the dbGaP database (Tryka et al., 2014). The *P-*values of the co-expression analysis were subjected to false discovery rate (FDR) adjustment. The cutoff values were PCC > 0.5 and FDR < 0.05.

### Construction of the AMCEN

We constructed the aberrantly methylated-differentially expressed genes associated ceRNA network (AMCEN) based on aberrantly methylated-differentially expressed mRNA–miRNA–lncRNA ceRNA triples using Cytoscape v3.8.1. The AMCEN for MG was constructed based on the theory that lncRNAs share common miRNA binding sites with mRNAs and function as ceRNAs to regulate mRNAs. For a given lncRNA–miRNA–mRNA interaction, both mRNA and lncRNA shared common miRNAs and were co-expressed for merging into a competing triplet. After identifying and assembling all lncRNA–miRNA–mRNA competing triplets by using cumulative hypergeometric test and PCC analysis, we constructed the AMCEN. The network was visualized using Cytoscape software, in which nodes represent miRNAs, genes and lncRNAs, and edges represent their interactions. Topologic features including degree and betweenness distribution were analyzed for all nodes in the AMCEN.

### Clinical Samples

A total of 27 patients with myasthenia gravis (MG group) admitted to the Second Affiliated Hospital of Harbin Medical University were selected as the experimental subjects. A total of 27 sex- and age- matched healthy subjects without autoimmune diseases and without any drug therapies were selected as the control group (Control group). According to the Chinese Guidelines for the Diagnosis and Treatment of Myasthenia Gravis (2015 Edition), MG patients should meet the following diagnostic criteria: first, patients should have the typical clinical characteristics of fluctuating fatigue of muscles, and in addition, it should meet any of the following three criteria: (i) pharmacological examination: positive for neostigmine test; (ii) Electrophysiological characteristics: repeated electrical nerve stimulation recorded complex muscle action and single fiber EMG recorded the neuromuscular junction for longer transmission; (iii) Serum antibody was positive. All the selected MG patients met the above diagnostic criteria and did not receive immunosuppressant treatment, and obtained informed consent of the selected patients. Peripheral blood samples were collected from each subject in tubes containing ethylenediaminetetraacetic acid, and PBMCs were isolated using lymphocyte separation medium. This study was approved by the Ethics Committee of The Second Affiliated Hospital of Harbin Medical University.

### Real-Time PCR Analysis

Total RNA was extracted from PBMCs using Trizol reagent (Sigma Life Science, Darmstadt, Germany) following the manufacturer’s instructions. Reverse transcription of total RNA into complementary DNA (cDNA) was performed using a Transcriptor First Strand cDNA Synthesis Kit (Roche, Switzerland) according to the manufacturer’s instructions. LINC00173 expression level was detected by quantitative real-time polymerase chain reaction (qRT-PCR) using FastStart Universal SYBR Green Master (Roche, Switzerland). The relative expression level was calculated using the 2^–ΔΔCt^ method using glyceraldehyde 3-phosphate dehydrogenase for normalization. The primer used for qRT-PCR was illustrated in Supplementary Table 1.

## Results

### Identification of DEGs and DMGs in MG

**FIGURE 1:**
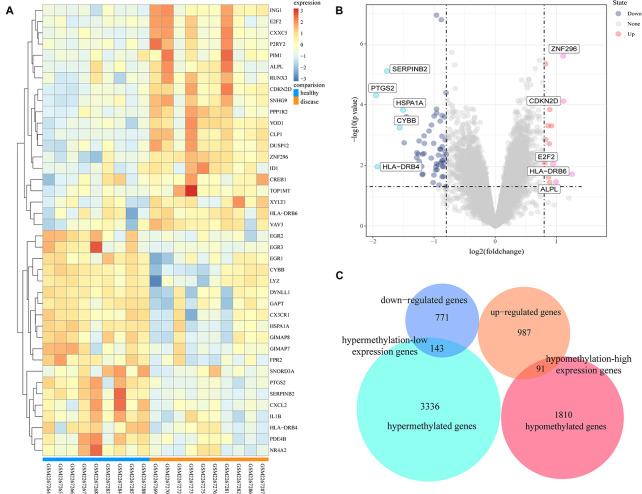
Analysis of differentially expressed genes and differentially methylated genes. **(A)** Representative heatmap of the top 40 differentially expressed genes in dataset GSE85452 (20 up-regulated genes and 20 down-regulated genes). **(B)** Representative volcanic map of differentially expressed genes distribution in GES85452. **(C)** Identification of aberrantly methylated-differentially expressed genes in gene expression dataset (GSE85452) and gene methylation dataset (GSE85647).

The GSE85452 and GSE85647 datasets were separately screened with GEO2R online software for DEGs or DMGs. In GSE85452, 987 DEGs were upregulated and 771 were downregulated and in GSE85647, 3336 genes were hypermethylated and 1810 were hypomethylated. A total of 143 hypermethylation-low expression genes were obtained by overlapping 3336 hypermethylated and 771 downregulated genes; and 91 hypomethylation-high expression genes were obtained by overlapping 1,810 hypomethylated and 987 upregulated genes (Figure 1).

### GO and KEGG Pathway Analyses of Aberrantly Methylated DEGs

**FIGURE 2:**
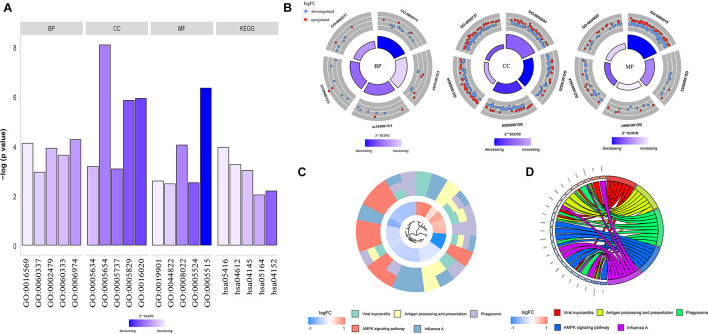
GO functional enrichment and KEGG pathway analysis of hypermethylation-low expression genes and hypomethylation-high expression genes. **(A)** Bar graph of BP, CC, MF and KEGG. **(B)** Distribution of hypermethylation-low expression genes and hypomethylation-high expression genes in each GO term of BP, CC, MF. Red represents hypermethylation-low expression genes; Blue represents hypomethylation-high expression genes. **(C)** KEGG pathway analysis of hypermethylation-low expression genes and hypomethylation-high expression genes. Different colors represent different pathways. **(D)** Genes distribution in different KEGG pathways.

To determine the functions of the identified hypermethylation-low expression and hypomethylation-high expression genes, GO and KEGG pathway analyses were carried out with DAVID. The top five significant GO terms and KEGG pathways are shown in Figure 2 and Table 1. Hypermethylation-low expression and hypomethylation-high expression genes were enriched in the biological processes of cellular response to DNA damage stimulus; covalent chromatin modification; antigen processing and presentation of exogenous peptide antigen via major histocompatibility complex (MHC) class I, TAP-dependent; IFN-γ-mediated signaling pathway; and type I IFN signaling pathway. For cellular component, the genes showed enrichment in nucleoplasm, membrane, cytosol, nucleus, and cytoplasm; and for molecular function, the genes were mostly enriched in protein binding. These results suggest that the identified genes play an important role in the pathogenesis of MG. Additionally, the KEGG pathway enrichment analysis showed that the genes were significantly enriched in viral myocarditis and antigen processing and presentation, indicating that the pathogenesis of MG is closely related to autoimmunity.

**TABLE 1:** GO and KEGG analysis of hypermethylation-low expression genes and hypomethylation-high expression genes.

Category	Term	Gene count	%	*P-*value
BP	GO:0006974∼cellular response to DNA damage stimulus	12	5.19	5.57E-05
BP	GO:0016569∼covalent chromatin modification	9	3.89	7.88E-05
BP	GO:0002479∼antigen processing and presentation of exogenous peptide antigen via MHC class I, TAP-dependent	7	3.03	1.24E-04
BP	GO:0060333∼interferon-gamma-mediated signaling pathway	7	3.03	2.40E-04
BP	GO:0060337∼type I interferon signaling pathway	6	2.59	1.15E-03
CC	GO:0005654∼nucleoplasm	68	29.43	8.45E-09
CC	GO:0016020∼membrane	53	22.94	1.21E-06
CC	GO:0005829∼cytosol	70	30.30	1.48E-06
CC	GO:0005634∼nucleus	89	38.52	6.72E-04
CC	GO:0005737∼cytoplasm	86	37.22	8.48E-04
MF	GO:0008022∼protein C-terminus binding	145	62.77	4.66E-07
MF	GO:0008022∼protein C-terminus binding	11	4.76	9.19E-05
MF	GO:0019901∼protein kinase binding	13	5.62	2.60E-03
MF	GO:0005524∼ATP binding	32	13.85	3.04E-03
MF	GO:0044822∼poly(A) RNA binding	26	11.25	3.40E-03
Pathway	hsa05416: Viral myocarditis	7	3.03	1.15E-04
Pathway	hsa04612: Antigen processing and presentation	7	3.03	5.62E-04
Pathway	hsa04145: Phagosome	9	3.89	9.68E-04
Pathway	hsa04152: AMPK signaling pathway	7	3.03	6.54E-03
Pathway	hsa05164: Influenza A	8	3.46	9.48E-03

*BP, biological process; MF, molecular function; CC, cellular component.*

### GSEA Enrichment in GSE85452

GSEA enrichment analysis showed that lipid droplet organization, chaperone binding, actin filaments, regulation of cellular amino acid metabolic process, negative regulation of BMP signaling pathway, endopeptidase complex, viral release from host cell, nucleobase containing compound kinase activity, protein demethylase activity had the largest NES, and the details were reported in Supplementary Figure 1 and Supplementary Table 2.

### Construction of a PPI Network, Module Analysis, and Hub Gene Selection

**FIGURE 3:**
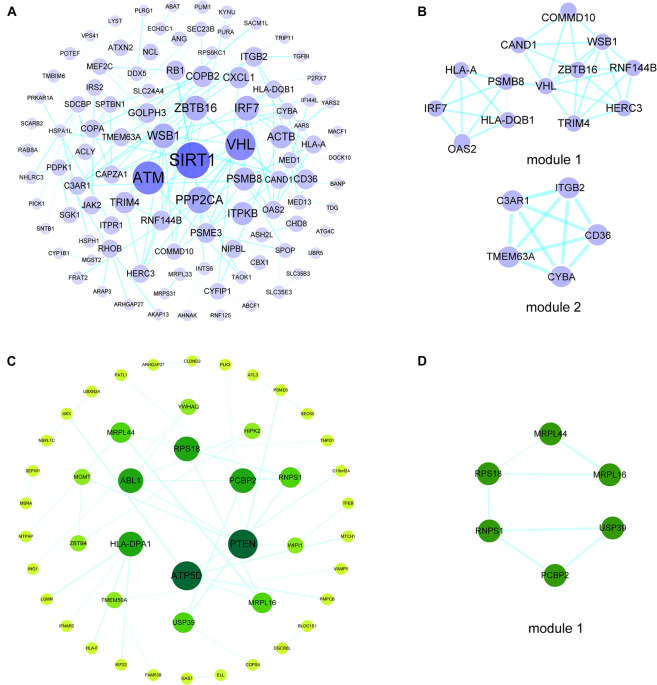
PPI networks and modules of hypermethylation-low expression genes and hypomethylation-high expression genes. **(A)** PPI network of hypermethylation-low expression genes. The bigger and darker the dot is, the higher degree the dot has. **(B)** Top two modules of hypermethylation-low expression genes PPI network. **(C)** PPI network of hypomethylation-high expression genes. The bigger and darker the dot is, the higher degree the dot has. **(D)** The top module of hypomethylation-high expression genes PPI network.

To investigate the interactions of hypermethylation-low expression or hypomethylation-high expression genes, we used the STRING database along with MCODE and cytoHubba to construct a PPI network and define the modules and hub genes (Figures 3A,B). The top 5 hub genes for the hypermethylation-low expression gene PPI network were Von Hippel–Lindau tumor suppressor (VHL), zinc finger and BTB domain-containing protein (ZBTB)16, WD repeat and SOCS box-containing protein (WSB)1, tripartite motif containing (TRIM)4, and ring finger protein (RNF)144B; and the top 5 hub genes in the hypomethylation-high expression gene PPI network were phosphatase and tensin homolog (PTEN), ATP synthase F1 subunit delta (ATP5D), Abelson tyrosine-protein kinase (ABL)1, poly(rC)-binding protein (PCBP)2, and human leukocyte antigen (HLA) class II histocompatibility antigen, DP alpha 1 chain (HLA-DPA1) (Figures 3C,D).

### AMCEN and Topologic Analysis

**FIGURE 4:**
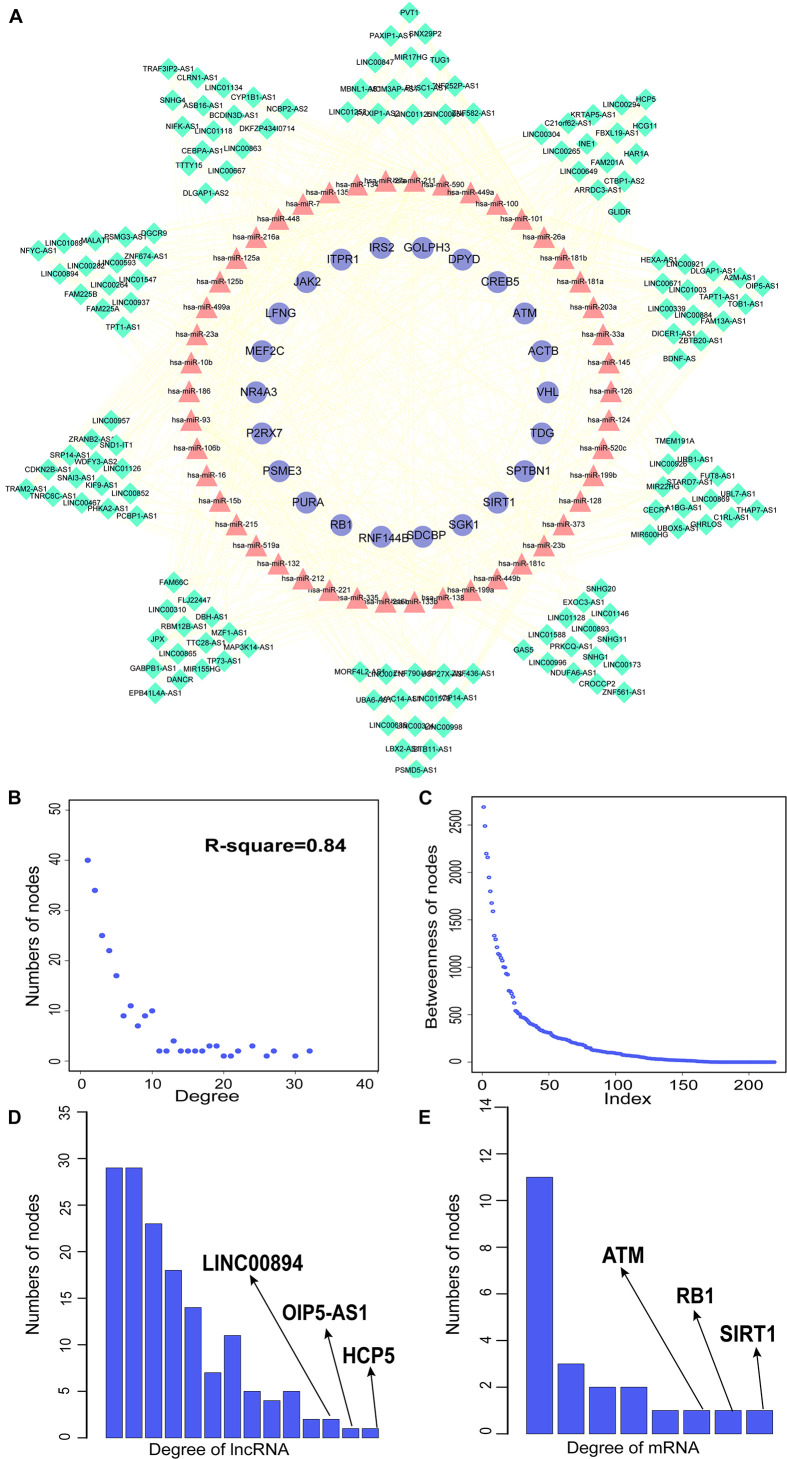
Construction of AMCEN by hypermethylation-low expression genes and topological property. **(A)** The ceRNA network. Purple circles represent mRNAs, red triangles represent miRNAs, green diamonds represent lncRNAs and lines represent their regulatory interactions. **(B)** The node degree distribution of the global ceRNA network. **(C)** The node betweenness distribution of the network. **(D)** Degree distribution of hypermethylation-low expression genes. **(E)** Degree distribution of lncRNAs.

**FIGURE 5:**
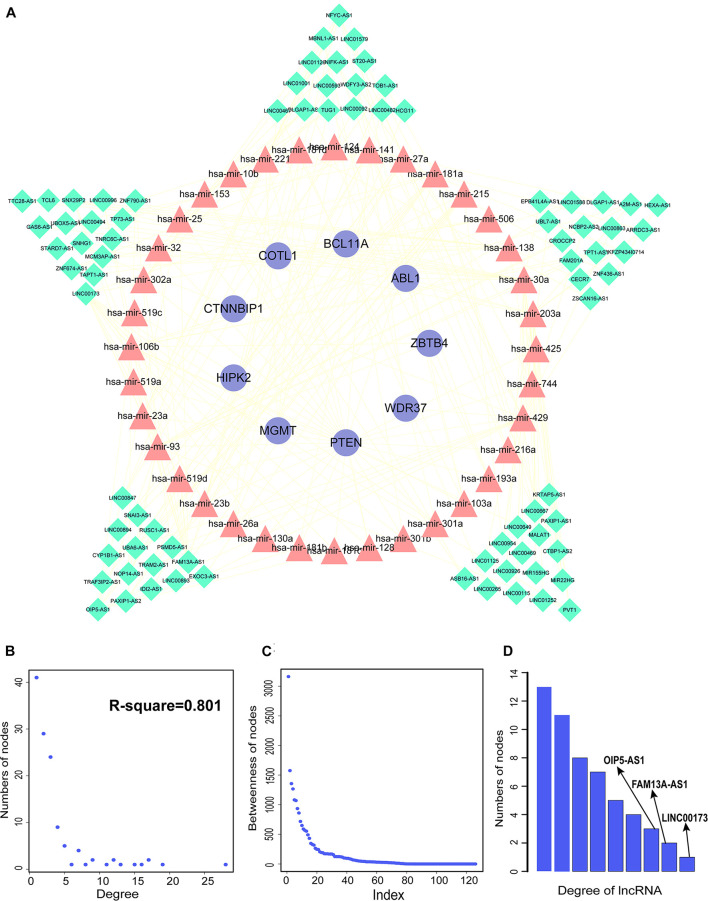
Construction of AMCEN by hypomethylation-high expression genes and topological property. **(A)** The ceRNA network. Purple circles represent mRNAs, red triangles represent miRNAs, green diamonds represent lncRNAs and lines represent their regulatory interactions. **(B)** The node degree distribution of the ceRNA network. **(C)** The node betweenness distribution of the network. **(D)** Degree distribution of lncRNAs.

We constructed aberrantly methylated gene-associated ceRNA networks by matching hypermethylation-low expression or hypomethylation-high expression genes with mRNA–miRNA and miRNA–lncRNA interactions and performing cumulative hypergeometric testing and lncRNA–mRNA co-expression analysis to screen triples for AMCEN construction (Figures 4, 5). We calculated the betweenness of the nodes in the AMCEN, with higher values reflecting a more important role in maintaining tight connectivity in the network. There were 744 mRNA–miRNA–lncRNA ceRNA triples of hypermethylation-low expression genes with 22 mRNAs, 46 miRNAs, and 151 lncRNAs (Figure 4); and 197 mRNA–miRNA–lncRNA ceRNA triples of hypomethylation-high expression genes with 9 mRNAs, 36 miRNAs, and 81 lncRNAs (Figure 5). In the hypermethylation-low expression gene-associated ceRNA network, sirtuin (SIRT)1 was the most important gene and the lncRNA HCP5 had the highest degree. In the hypomethylation-high expression gene-associated ceRNA network, PTEN was the most important gene and the lncRNA LINC00173 had the highest degree.

**FIGURE 6:**
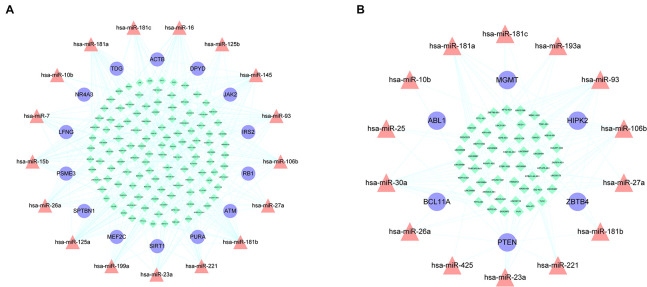
AMCENs after matching miRNAs. **(A)** Hypermethylation-low expression genes mediated ceRNA network. Purple circles represent mRNAs, red triangles represent miRNAs, green diamonds represent lncRNAs and lines represent their regulatory interactions. **(B)** Hypomethylation-high expression genes mediated ceRNA network. Purple circles represent mRNAs, red triangles represent miRNAs, green diamonds represent lncRNAs and lines represent their regulatory interactions.

miRNAs are a type of ceRNA that play an important role in ceRNA networks. We manually selected 192 MG risk miRNAs to increase the reliability of our analysis and overlapped these with aberrantly methylated ceRNA interactions. This yielded a hypermethylation-low expression gene-associated ceRNA network comprising 333 mRNA–miRNA–lncRNA ceRNA triples including 14 genes, 17 miRNAs, and 122 lncRNAs (Figure 6A); and a hypomethylation-high expression gene-associated ceRNA network comprising 97 mRNA–miRNA–lncRNA ceRNA triples including 6 genes, 14 miRNAs, and 49 lncRNAs (Figure 6B). These aberrantly methylated DEGs and ceRNA networks are presumed to be involved in the pathogenesis of MG.

### Identification of Biomarkers Based on Aberrantly Methylated-Differentially Expressed Genes and ceRNA Networks

By matching genes in ceRNA networks and hub genes of the MG PPI network, we identified miRNAs that interact with PTEN including hsa-miR-106b, hsa-miR-10b, hsa-miR-181a, hsa-miR-181b, hsa-miR-181c, hsa-miR-193a, hsa-miR-221, hsa-miR-23a, hsa-miR-25, hsa-miR-26a, hsa-miR-425, and hsa-miR-93. The lncRNAs co-expressed with PTEN in the ceRNA networks were LINC00173, family with sequence similarity 13, antisense RNA A1 (FAM13A-AS1), ankyrin repeat and SOCS box-containing antisense RNA (ASB16-AS)1, Opa-interacting protein 5 antisense RNA (OIP5-AS)1, transducer of ERBB2 antisense RNA (TOB1-AS)1, LINC01126, nucleolar protein interacting with the FHA domain of MKI67 antisense RNA (NIFK-AS)1, LINC01001, muscleblind-like splicing regulator antisense RNA (MBNL1-AS)1, nuclear transcription factor Y subunit gamma antisense RNA (NFYC-AS)1, taurine upregulated gene (TUG)1, LINC00593, DLG-associated protein 1 antisense RNA (DLGAP1-AS)2, HLA complex group (HCG)11, LINC00467, and WD repeat and FYVE domain-containing 3 antisense RNA (WDFY3-AS)2. The miRNA hsa-miR-30a combined with ABL1 in the ceRNA networks; the co-expressed lncRNAs were erythrocyte membrane protein band 4.1-like 4A antisense RNA (EPB41L4A-AS)1, small nucleolar RNA host gene (SNHG)1, sorting nexin 29 pseudogene (SNX29P)2, C terminal-binding protein antisense RNA (CTBP1-AS)2, plasmacytoma variant translocation (PVT)1, metastasis-associated lung adenocarcinoma transcript (MALAT)1, miR-22 host gene (MIR22HG), U-bx domain-containing 5 antisense RNA (UBOX5-AS)1, StAR-related lipid transfer domain-containing 7 antisense 1 (STARD7-AS1), LINC00115, keratin-associated protein 5-5 antisense RNA (KRTAP5-AS)1, tumor protein 73 antisense RNA (TP73-AS)1, LINC00469, LINC00926, and LINC00667. Based on the degree of lncRNAs in ceRNA networks, the lncRNAs LINC00173, FAM13A-AS1, and OIP5-AS1 were found to be associated with PTEN in ceRNA networks (Table 2). Thus, PTEN and ABL1 are critical genes in the pathogenesis of MG and the lncRNAs LINC00173, FAM13A-AS1, and OIP5-AS1 are potential disease biomarkers.

**TABLE 2:** ceRNAs associated with PTEN.

Gene	miRNA	lncRNA
PTEN	hsa-miR-106b	FAM13A-AS1
PTEN	hsa-miR-23a	FAM13A-AS1
PTEN	hsa-miR-25	FAM13A-AS1
PTEN	hsa-miR-93	FAM13A-AS1
PTEN	hsa-miR-106b	LINC00173
PTEN	hsa-miR-181a	LINC00173
PTEN	hsa-miR-181b	LINC00173
PTEN	hsa-miR-181c	LINC00173
PTEN	hsa-miR-23a	LINC00173
PTEN	hsa-miR-93	LINC00173
PTEN	hsa-miR-181a	OIP5-AS1
PTEN	hsa-miR-181b	OIP5-AS1
PTEN	hsa-miR-181c	OIP5-AS1
PTEN	hsa-miR-25	OIP5-AS1
PTEN	hsa-miR-93	OIP5-AS1

### Validation of LINC00173 Expression

**FIGURE 7:**
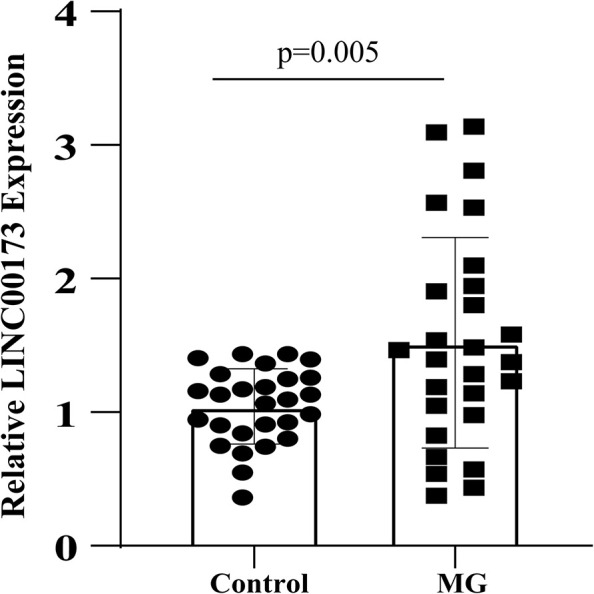
LINC00173 expression in MG. LINC00173 was up regulated in PBMCs from MG patients compared with healthy controls.

The expression level of LINC00173 was evaluated by qRT-PCR in PBMCs of MG patients and control subjects. LINC00173 was upregulated in MG patients compared with controls (*P* = 0.005), which indicated LINC00173 might be a potential biomarker for MG (Figure 7).

## Discussion

MG is an autoantibody-mediated disease of the nervous system with an ill-defined etiology. Because of the complex pathogenesis and heterogeneity of MG, there is no single treatment that can be applied to all MG patients (Sanders et al., 2016). DNA methylation marks can serve as a biomarker for MG (Fang et al., 2018). The discovery of ceRNA networks has provided a basis for investigating the molecular mechanisms of MG, which could aid diagnosis, treatment, and prognostic assessment. In this study, we identified 143 hypermethylation-low expression and 91 hypomethylation-high expression genes by analyzing microarray-based gene expression and gene methylation data for MG. DEGs and DMGs reflected the genes and pathways modulated by aberrant methylation in MG. We then constructed an AMCEN to determine the main factors contributing to MG pathogenesis.

Hypermethylation-low expression and hypomethylation-high expression genes in MG were enriched in biological processes of cellular response to DNA damage stimulus; covalent chromatin modification; antigen processing and presentation of exogenous peptide antigen via MHC class I, TAP-dependent; IFN-γ–mediated signaling pathway; and type I IFN signaling pathway. As an autoimmune disease, MG is caused by both genetic and external factors (Meriggioli and Sanders, 2009). Genetics can influence disease susceptibility, which can be modulated by external factors such as infection or diet (Gilhus and Verschuuren, 2015) that lead to chromatin modification. Infections can induce the production of antibodies that act as autoantibodies against AChR; in fact, exacerbation of MG is often associated with infection (Hehir and Silvestri, 2018). Thus, aberrant antigen processing and presentation may contribute to the pathogenesis of MG. IFN-γ level was shown to be elevated in MG patients (Link et al., 1994). Viral infection and an increased level of type I IFN have been linked to thymic changes similar to those observed in MG patients with anti-AChR antibodies (Berrih-Aknin and Le Panse, 2014). Consistent with a viral etiology for MG and the role of immune responses, the results of the KEGG pathway analysis revealed significant enrichment of the viral myocarditis and antigen processing and presentation pathways.

The top 5 hub genes in the PPI network of hypermethylation-low expression genes were VHL, ZBTB16, WSB1, TRIM4, and RNF144b. VHL disease is caused by mutation of the VHL gene on chromosome 3p25-26 (Kaelin, 2007). A case was reported of an antibody-positive MG patient with thymoma concurrent with VHL (Sheth et al., 2005). Although this was a unique case, it suggests a potential genetic link between MG and VHL. ZBTB16, also known as promyelocytic leukemia zinc finger (PLZF), is a transcription factor that promotes the recruitment of effector T helper cells during the development of innate lymphocyte lineages and is also essential for the development of osteoblasts and spermatogonia (Mao et al., 2017). Another study found that DNA methylation in the promoter region of the ZBTB16 gene may regulate gene expression during osteoblast differentiation (Marofi et al., 2019). Thus, ZBTB16 may contribute to abnormal T cell development in MG, and ZBTB16 methylation is a potential mechanism of MG pathogenesis. WSB1, TRIM4, and RNF144b are associated with multiple cancers including neuroblastoma (Shichrur et al., 2014), chordoma (Wang C. B. et al., 2020) and hepatocellular carcinoma (Dong et al., 2020). Abnormal methylation of TRIM4 in immune pathways may be associated with neural tube defects (Zhang et al., 2019). RNF144B is thought to be involved in the development of chordoma through a ceRNA network (Wang C. B. et al., 2020). In the PPI network of hypomethylation-high expression genes, the top 5 hub genes were PTEN, ATP5D, ABL1, PCBP2, and HLA-DPA1. PTEN is a tumor suppressor and regulator of metabolism (Chen et al., 2018) that is expressed in some types of thymoma, although methylation of the gene has not been detected in thymoma samples (Masunaga et al., 2017). ATP5D was shown to be differentially expressed in amyotrophic lateral sclerosis patients and was linked to mitochondrial dysfunction in synaptic clefts (Engelen-Lee et al., 2017). ABL1 and PCBP2 are associated with immunity (Shi et al., 2014; Rafei et al., 2019), and HLA-DPA1 has been implicated in juvenile-onset MG (Feng et al., 2019). Elucidating the functions of these genes may provide new insights into MG pathogenesis.

PTEN and ABL1 were found to be components of hypermethylation-low expression and hypomethylation-high expression gene-associated ceRNA networks. PTEN was involved in 33 mRNA–miRNA–lncRNA triples. Previous study had found that LINC00173 promotes the proliferation and migration of vascular endothelial cells in lung squamous cell carcinoma by acting as ceRNA to bind with miR-511-5p and regulate VEGFA expression (Chen et al., 2020). LINC00173, which had the highest node degree in the hypomethylation-high expression gene-associated ceRNA network, has been investigated as part of the ceRNA networks of lung cancer (Chen et al., 2020), breast cancer (Fan et al., 2020), and glioma (Du et al., 2020), but its molecular mechanism in autoimmune diseases of the nervous system was still unclear. As a constituent of one of the 15 ABL1-associated ceRNA triples, the lncRNA MALAT1 was shown to be downregulated in MG patients and to act as a ceRNA for miR-338-3p (Kong et al., 2019). In the hypermethylation-low expression gene-associated ceRNA network, SIRT1 had the highest node degree. SIRT1 has been linked to immune-related diseases such as tumors and autoimmune diseases (Yu et al., 2018) and was decreased in the PBMCs of multiple sclerosis patients during relapses (Martin et al., 2015). The lncRNAs HCP5, OIP5-AS1, and LINC00894 were the most important lncRNAs in the ceRNA network. HCP5 is associated with several autoimmune diseases including psoriatic arthritis (Liu et al., 2008), systemic lupus erythematosus (Ciccacci et al., 2014), and Graves’ disease (Lane et al., 2020); and OIP5-AS1 was found to play a critical role in the ceRNA network of multiple sclerosis (Ding et al., 2021). LINC00894 may be related to drug resistance in breast cancer (Zhang X. et al., 2018). NR4A3, which is associated with MG (Bernasconi et al., 2003), was involved in 3 ceRNA triples; one of these includes zinc finger 674 antisense RNA (ZNF674-AS)1, which has been investigated in relation to cancers. Little is known about the functions of VAC14-AS1 and SNX29P2.

This study had several limitations. Firstly, lncRNAs and the ceRNA hypothesis have not been extensively investigated in MG, so most lncRNAs that have not been previously reported in this context and require validation. Secondly, the DEGs and aberrantly methylated genes were screened from an online database, and the relatively small number of samples reduced the reliability of the data. Finally, we constructed the AMCEN and identified several critical ceRNA triples, but the actual relationships between their components need to be verified experimentally.

## Conclusion

In Conclusions, we identified 143 hypermethylation-low expression and 91 hypomethylation-high expression genes by overlapping DEGs and DMGs. These genes were mainly enriched in chromatin modification and immune process. Phosphatase and tensin homolog (PTEN) and Abelson tyrosine-protein kinase (ABL)1 were critical genes in both PPI networks and ceRNA networks. This revealed that PTEN and ABL1 were critical genes while the lncRNAs LINC00173, FAM13A-AS1, and OIP5-AS1 were potential biomarkers in MG. LINC00173 was validated to be upregulated in MG patients compared with controls. These findings provide insight into the molecular pathogenesis of MG as well as novel therapeutic targets for disease treatment.

## Data Availability Statement

The original contributions presented in the study are included in the article/Supplementary Material, further inquiries can be directed to the corresponding author/s.

## Ethics Statement

The studies involving human participants were reviewed and approved by the Ethics Committee of The Second Affiliated Hospital of Harbin Medical University. The patients/participants provided their written informed consent to participate in this study.

## Author Contributions

SX, JW, and LW conceived, designed the study, and revised the manuscript. SX, TW, XL, and HZ collected the data. LL, XK, and SL were performed the bioinformatics analysis. SX, XW, and HG analyzed and visualized the results. SX and LL conducted the experiments. SX performed the statistical analyses. SX and TW wrote the manuscript. All authors read, edited and approved the final manuscript.

## Conflict of Interest

The authors declare that the research was conducted in the absence of any commercial or financial relationships that could be construed as a potential conflict of interest.

## Publisher’s Note

All claims expressed in this article are solely those of the authors and do not necessarily represent those of their affiliated organizations, or those of the publisher, the editors and the reviewers. Any product that may be evaluated in this article, or claim that may be made by its manufacturer, is not guaranteed or endorsed by the publisher.

## Footnotes

1
https://www.ncbi.nlm.nih.gov/geo/

2
http://www.ncbi.nlm.nih.gov/pubmed

3
https://www.ncbi.nlm.nih.gov/geo/geo2r/

4
https://david.ncifcrf.gov/

5
http://string.embl.de/

6
http://www.cytoscape.org/

## Supplementary Material

Supplementary Figure 1 Gene Set Enrichment Analysis (GSEA) of mRNAs in GSE85452.
