# Supplementary figures and images for: Identification of LINC00173 in Myasthenia Gravis by Integration Analysis of Aberrantly Methylated- Differentially Expressed Genes and ceRNA Networks
Source: Front Genet. 2021 Sep 16;12:726751. doi: 10.3389/fgene.2021.726751 (PMC8481885; doi:10.3389/fgene.2021.726751)

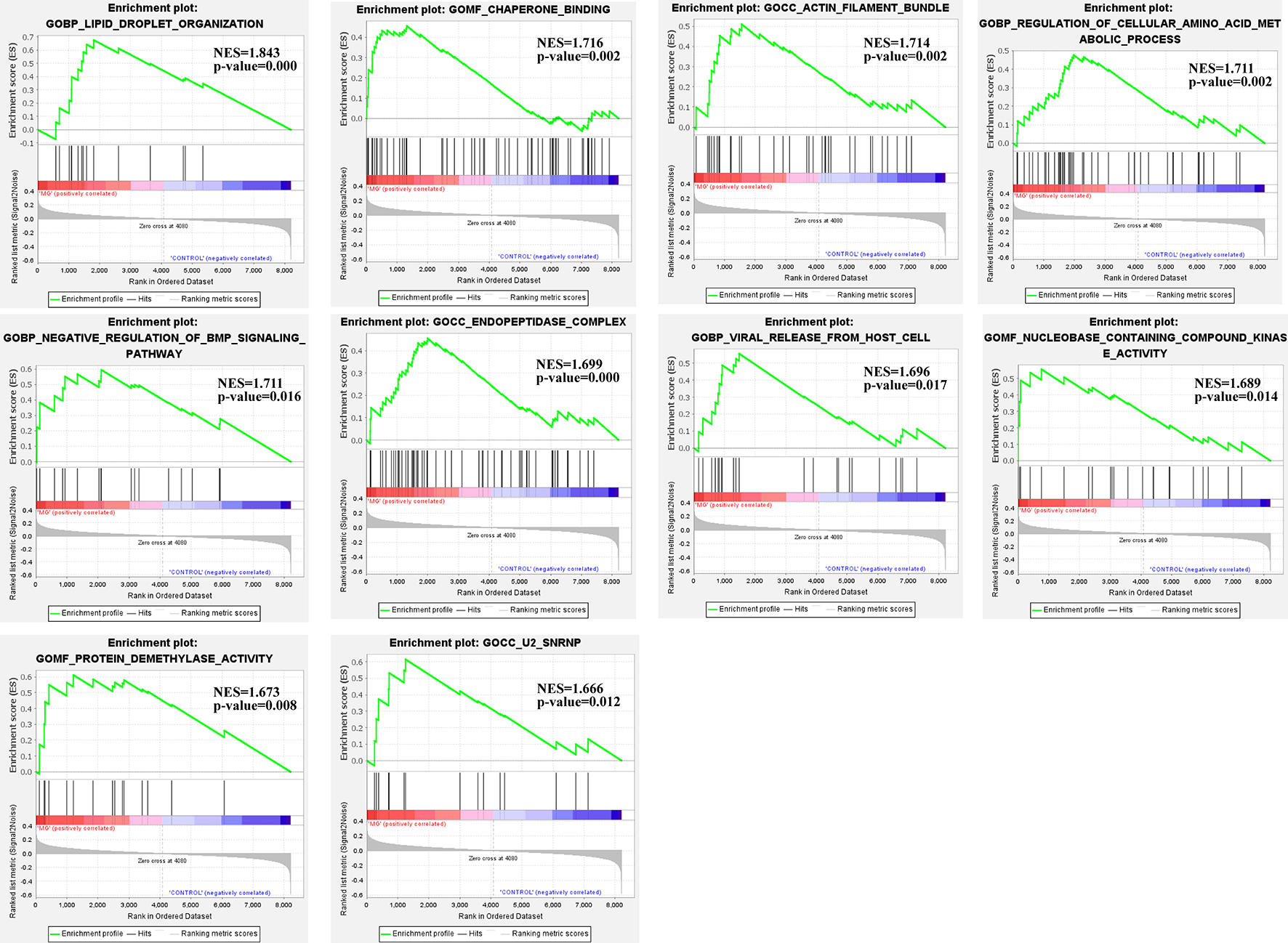

Supplement: Supplementary Figure 1 — Gene Set Enrichment Analysis (GSEA) of mRNAs in GSE85452. [file Image_1.tif]
