# Supplementary material for: Identification of LINC00173 in Myasthenia Gravis by Integration Analysis of Aberrantly Methylated- Differentially Expressed Genes and ceRNA Networks
Source: Front Genet. 2021 Sep 16;12:726751. doi: 10.3389/fgene.2021.726751 (PMC8481885; doi:10.3389/fgene.2021.726751)
Supplement: Supplementary file 2 [file Table_1.DOCX]

**Table S1 Primer used for qRT‐PCR**

| **Name** |  | **Sequences** |
| --- | --- | --- |
| LINC00173 | Forward primer (5′‐>3′) | GGCTTTATTTATGGTGTTACGA |
|  | Reverse primer (5′‐>3′) | CGATTCTGGGACCTGTGG |
