# Supplementary material for: Identification of LINC00173 in Myasthenia Gravis by Integration Analysis of Aberrantly Methylated- Differentially Expressed Genes and ceRNA Networks
Source: Front Genet. 2021 Sep 16;12:726751. doi: 10.3389/fgene.2021.726751 (PMC8481885; doi:10.3389/fgene.2021.726751)
Supplement: Supplementary file 3 [file Table_2.DOCX]

**Table S2 Top 10 gene sets of GSEA**

| **NAME** | **ES** | **NES** | **p-value** |
| --- | --- | --- | --- |
| GOBP_LIPID_DROPLET_ORGANIZATION | 0.675837 | 1.843581 | 0 |
| GOMF_CHAPERONE_BINDING | 0.456046 | 1.7160896 | 0.002283105 |
| GOCC_ACTIN_FILAMENT_BUNDLE | 0.51071 | 1.7147163 | 0.002118644 |
| GOBP_REGULATION_OF_CELLULAR_AMINO_ACID_METABOLIC_PROCESS | 0.476573 | 1.711488 | 0.002057613 |
| GOBP_NEGATIVE_REGULATION_OF_BMP_SIGNALING_PATHWAY | 0.594427 | 1.7110909 | 0.016 |
| GOCC_ENDOPEPTIDASE_COMPLEX | 0.456185 | 1.6993772 | 0 |
| GOBP_VIRAL_RELEASE_FROM_HOST_CELL | 0.555417 | 1.6966022 | 0.017021276 |
| GOMF_NUCLEOBASE_CONTAINING_COMPOUND_KINASE_ACTIVITY | 0.557593 | 1.6891369 | 0.01443299 |
| GOMF_PROTEIN_DEMETHYLASE_ACTIVITY | 0.612811 | 1.6739267 | 0.008474576 |
| GOCC_U2_SNRNP | 0.615355 | 1.666763 | 0.012578616 |
